# ECG Changes in a Case of Posterior Myocardial Infarction in the Presence of Right Bundle Branch Block

**DOI:** 10.7759/cureus.13281

**Published:** 2021-02-11

**Authors:** Aliya Ramjaun, Ankit Garg, Marlee Klaiman, Matthew Sibbald, Junghwan (Kevin) Dong

**Affiliations:** 1 Emergency Medicine, McMaster University, Hamilton, CAN; 2 Cardiology, McMaster University, Hamilton, CAN

**Keywords:** cardiac electrophysiology, emergency medicine, cardiology, myocardial infarction

## Abstract

A 70-year-old male with hypertension and diabetes presented to the emergency department with a 1-hour history of chest pain. Initial 12-lead ECG revealed a right bundle branch block (RBBB) and ST depression (STD) in V2-V4. The anterior STD prompted a 15-lead ECG in which there was no evidence of ST elevation (STE). With a positive troponin, cardiology was consulted and the patient was admitted as a high-risk non-ST-elevation myocardial infarction (NSTEMI). Subsequently, his chest pain returned without further ST changes, regardless the patient went for emergency coronary angiography, which found a complete occlusion of the left circumflex artery. Anterior STD is a normal finding in RBBB and posterior STEs in the posterior leads are not always present making the recognition of posterior STEMI difficult. This case highlights three findings in leads V1-V3 that are concerning for posterior ischemia in the context of chest pain and an RBBB: tall R waves, upright T waves, and marked STD > 2 mm. This should prompt serial 15-lead ECGs and prompt cardiology consultation.

## Introduction

The diagnosis of acute myocardial infarction (MI) secondary to left circumflex artery occlusion is difficult to make, largely due to the insensitivity of a standard 12-lead ECG. Even with a 15-lead ECG, posterior MI cases are the most commonly missed [1]. This is further complicated when a right bundle branch block (RBBB) is present, as ST depressions (STDs) in the anterior leads can be a normal finding. This case illustrates three subtle ECG findings that, in the context of ischemic symptoms and RBBB, should raise suspicion for posterior ischemia.

## Case presentation

**Figure 1:**
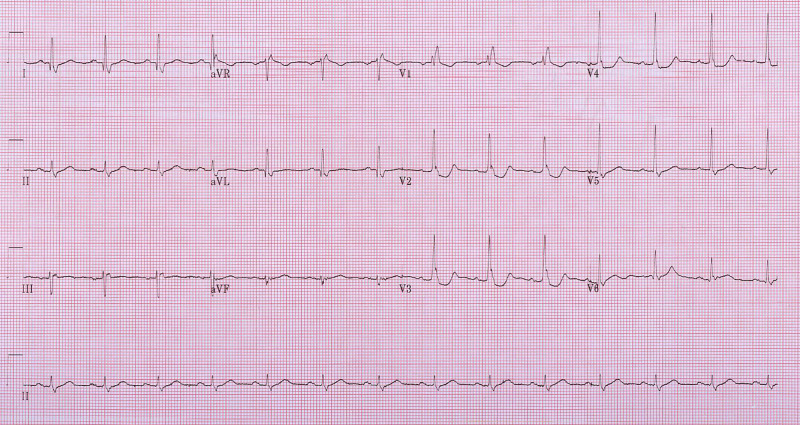
Twelve-lead ECG demonstrating ST depression in V2-V4 and RBBB with rSR’ in V1. RBBB - Right bundle branch block

**Figure 2:**
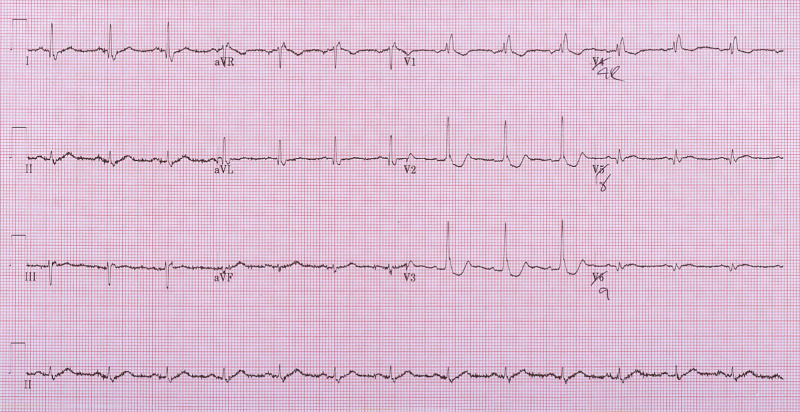
Fifteen-lead ECG demonstrating potential ST elevation but definite convex morphology in V8-V9.

A 70-year-old male presented to our emergency department (ED) with acute onset of 10/10 chest heaviness and pain radiating to the neck and left arm. This chest pain began at rest, one hour prior to his presentation. He was diaphoretic and fatigued at the time of onset. While en-route to the ED, he received 162 mg of aspirin. His past medical history included type 2 diabetes, hypertension, psoriatic arthritis, and prostate cancer (resected). His initial ECG did not demonstrate an ST-segment elevation MI (STEMI); however, suspicious ST changes in the right precordial anterior leads (Figure 1) prompted the acquisition of a 15-lead ECG (Figure 2).

The initial ECG revealed a right bundle branch block (RBBB) in V1 with T-wave inversion, and marked STD in the anterior leads V2-V4, without evidence for of ST elevation (STE) in the inferior leads. The 15-lead ECG also did not show clear evidence for STE (<0.5 mm) in leads V7, V8, or V9. With no evidence of shock and hypotension, 0.4 mg nitroglycerin was administered sublingually. With this, his symptoms improved. With an initial troponin rise of 330 ng/L, however, cardiology was consulted. As his chest pain initially resolved and because ECG diagnostic criteria were not met for posterior STEMI, cardiac catheterization was scheduled for the subsequent day and he was admitted for high-risk non-STEMI. However, within an hour, his symptoms recurred. Although another serial 15-lead ECG was done, it did not show any significant change. However, due to his clinical status, high-risk medical history, and persistent pain, emergent cardiac catheterization was carried out.

**Figure 3:**
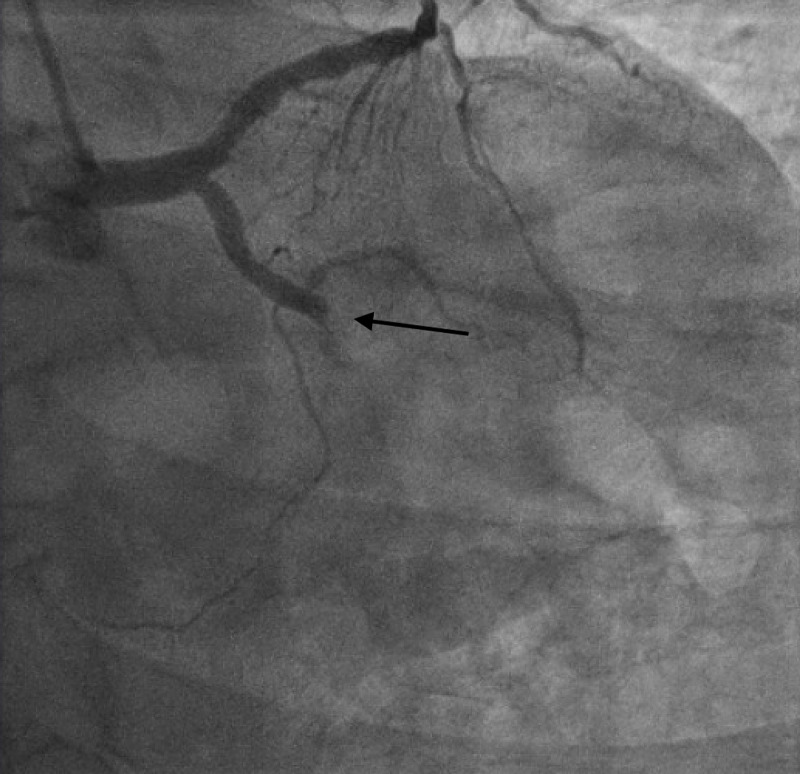
Left circumflex culprit lesion identified through coronary catheterization.

Cardiac catheterization revealed a culprit circumflex complete occlusion with thrombolysis in MI 0 (TIMI 0) flow (Figure 3), subsequently treated with a drug-eluting stent (DES). This was deemed to be acutely occluded and responsible for the patient's presentation. There was also diffuse disease in the dominant right coronary artery (RCA), left anterior descending (LAD) artery, and a focal proximal RCA lesion for which staged percutaneous coronary intervention was performed. Echocardiography demonstrated mild left ventricular dysfunction with apical lateral akinesis. The patient was discharged home, with the residual disease to be managed medically.

## Discussion

Acute posterior MI accounts for up to 20% of all MIs [1]. However, identification of posterior MI and electrically silent circumflex occlusion continues to be a diagnostic challenge, with cases often missed [1].

It has been well established that STD in the anterior leads V1-V3 is suggestive of posterior MI. This should prompt acquisition of a 15-lead ECG to assess the posterior myocardium, as V7-V9 are more specific for posterior MI. However, RBBB is commonly associated with STD in V1-V3 with inverted T waves, complicating the diagnosis of posterior MI in the setting of RBBB. In our case, despite the presence of RBBB, there are several subtle changes that, coupled with a concerning clinical presentation, made for a diagnosis of posterior MI, where typical 15-lead ECG changes alone were not compelling.

First, the presence of tall R waves in V2-V3 preceding R’ is abnormal in RBBB as the early depolarization vector in RBBB typically reflects normal left ventricular depolarization. In this case, the tall R waves in V2-V3 likely represent large posterior Q waves suggestive of transmural infarction [2]. In addition, typical RBBB is manifested by discordant T-wave inversions in V1-V3. In the ECGs presented, upright T waves in the context of tall R waves in the right precordial leads are highly suggestive of posterior MI [2,3]. Further, there is marked STD (>2 mm) in leads V2-V3, out of proportion to what is typically observed in RBBB. In the setting of chest pain, this should prompt the clinician to consider posterior ischemia [2,3].

Clinicians must also be aware of the difference in voltage criteria for posterior MI. As demonstrated by Wung and Drew, as little as 53% of posterior MIs resulting from circumflex occlusions produce an STE of at least 1 mm in posterior leads [4]. However, the detection of posterior MI increases to 94% when the STE threshold is reduced to 0.5 mm with 6% still being missed [4]. In the absence of meeting the above diagnostic ECG criteria in a patient with a suspicious clinical presentation, serial ECGs should be performed.

Where cardiac catheterization is not immediately available, fibrinolytic therapy may be appropriate if there is marked STD confined to V1-V4, accompanied by tall R waves in the right precordial leads and upright T waves. These ECG findings are indicative of true posterior MI with circumflex coronary occlusion (Class IIa Indication) [5]. If fibrinolysis is being considered, a 15-lead ECG with posterior (V7-V9) lead evaluation may help in providing additional diagnostic certainty as this improves specificity in detecting posterior MI [5].

## Conclusions

This case raises a number of important points in evaluating patients for posterior MI in the presence of RBBB. Specifically, STD in leads V1-V3 with a concerning clinical history should raise suspicion of a posterior STEMI. And, while discordant STD can be seen with RBBB, tall R waves preceding R’ in leads V2-V3 with upright T waves is abnormal and may suggest posterior ischemia. Finally, clinicians should be aware that while a posterior ECG with 0.5 mm STE increases sensitivity, clinical judgment should be used to increase detection of electrically silent posterior MIs.
